# Deep Sequencing of Distinct Preparations of the Live Attenuated Varicella-Zoster Virus Vaccine Reveals a Conserved Core of Attenuating Single-Nucleotide Polymorphisms

**DOI:** 10.1128/JVI.00998-16

**Published:** 2016-09-12

**Authors:** Daniel P. Depledge, Koichi Yamanishi, Yasuyuki Gomi, Anne A. Gershon, Judith Breuer

**Affiliations:** a MRC Centre for Medical Molecular Virology, Division of Infection and Immunity, London, United Kingdom; b The Research Foundation for Microbial Diseases of Osaka University, Biken, Japan; c Division of Infectious Disease, Columbia University Medical Center, New York, New York, USA; Oregon Health and Science University

## Abstract

The continued success of the live attenuated varicella-zoster virus vaccine in preventing varicella-zoster and herpes zoster is well documented, as are many of the mutations that contribute to the attenuation of the vOka virus for replication in skin. At least three different preparations of vOka are marketed. Here, we show using deep sequencing of seven batches of vOka vaccine (including ZostaVax, VariVax, VarilRix, and the Oka/Biken working seed) from three different manufacturers (VariVax, GSK, and Biken) that 137 single-nucleotide polymorphism (SNP) mutations are present in all vaccine batches. This includes six sites at which the vaccine allele is fixed or near fixation, which we speculate are likely to be important for attenuation. We also show that despite differences in the vaccine populations between preparations, batch-to-batch variation is minimal, as is the number and frequency of mutations unique to individual batches. This suggests that the vaccine manufacturing processes are not introducing new mutations and that, notwithstanding the mixture of variants present, VZV live vaccines are extremely stable.

**IMPORTANCE** The continued success of vaccinations to prevent chickenpox and shingles, combined with the extremely low incidence of adverse reactions, indicates the quality of these vaccines. The vaccine itself is comprised of a heterogeneous live attenuated virus population and thus requires deep-sequencing technologies to explore the differences and similarities in the virus populations between different preparations and batches of the vaccines. Our data demonstrate minimal variation between batches, an important safety feature, and provide new insights into the extent of the mutations present in this attenuated virus.

## INTRODUCTION

Primary infection with the varicella-zoster virus (VZV) causes chickenpox (varicella), during which the virus also establishes lifelong latency in sensory ganglia (reviewed in reference 1). Reactivation of the virus causes herpes zoster in up to 30% of infected individuals and carries the potential for a range of serious complications including postherpetic neuralgia, stroke, keratitis, and meningoencephalitis (2–5).

**FIG 1:**
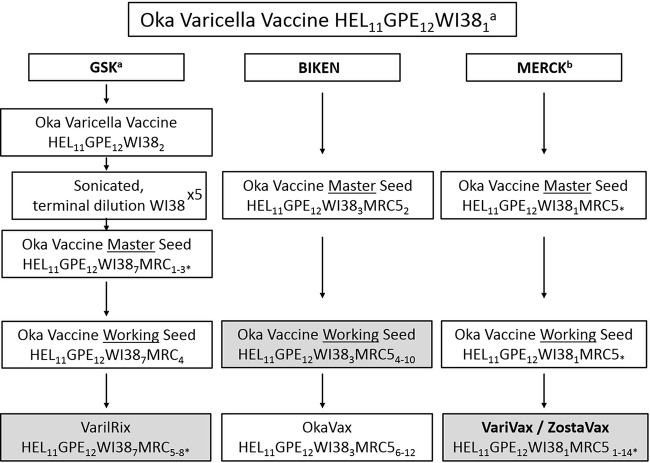
Overview of varicella vaccine production. Shaded cells indicate the preparations obtained and used in this study. HEL, human embryonic lung cell cultures; GPE, embryonic guinea pig cell cultures; WI38, human diploid cell cultures; MRC5, human diploid cell cultures. Subscript numbers indicate passage levels. Superscripts: a, information was obtained from a previous study (8); b, information obtained from Merck Product information IPC-VRV3-I-032008. *, Unknown number of passage levels in MRC5 cells. Where passage levels are given next to an asterisk (*), these have been inferred from superscript sources “a” and “b” above. World Health Organization guidelines indicate a limit for Oka strain varicella vaccine at passage level 38; hence, the final levels of passage in MRC5 cells can be estimated (8).

Since 1995, a live attenuated VZV vaccine (vOka) has been routinely administered to healthy children aged 12 to 18 months in the United States with significant success (6). Similar programs have since been established in a number of other countries, including Canada, Germany, Greece, Uruguay, and Australia (reviewed in reference 1). The most common VZV (vOka) vaccine preparations for the prevention of varicella are VarilRix (GSK), VariVax (Merck), and OkaVax(Biken), while a high-dose preparation (19,000 PFU versus 1,350 PFU) called ZostaVax (Merck) is routinely used to prevent episodes of herpes zoster. The vaccine itself was derived, by attenuation, from a wild-type virus (pOka) isolated from a small child (Oka) with primary varicella. Attenuation was achieved through serial passage in human embryo fibroblasts, guinea pig embryo fibroblasts, and WI-38 human diploid cells to generate the vOka/Biken seed virus (7). At least three vaccine preparations derived from this seed stock are currently in use (Fig. 1) (8). A comparative sequence analysis, using overlapping PCR and Sanger sequencing, of pOka and a batch of the Biken varicella vaccine (lot V-65, made in late 1990s) revealed 42 single-nucleotide polymorphism (SNP) differences between the wild-type and attenuated Oka strains, as well as variations in the lengths of the small repetitive R1, R3, R4, and OriS regions of the genome (9). Furthermore, this study established that the vOka seed comprises a heterogeneous population of vOka haplotypes. The SNP(s) responsible for attenuation has not been completely worked out, although current data suggest that the fixed vaccine SNP at position 106262 (R958G, ORF62), a very high frequency SNP at position 107252 (S628G, ORF62), and a third SNP at position 562 (*130R, ORF0) are important (9–17). In the context of a viral population, we are using the term SNP to indicate sites at which either (i) two or more alleles are present within that population or (ii) the fixed allele at a site differs from the parental sequence.

By analyzing vaccine allele frequencies at limited a subset of SNPs, we and others have observed that the vaccine allele frequency is lower in VariVax batches than VarilRix batches (18–20). More recently, deep sequencing of Merck VariVax vaccine batches using Roche 454 (21) and Illumina technologies (22) has identified more than 100 new variant sites, some unique to a single VariVax preparation and some common to multiple VariVax preparations. For most but not all of the new sites, the vaccine allele frequency is typically below 20%. However, it remains unclear how many of these sites are shared by different vaccine preparations (i.e., which sites are also present in Oka/Biken and GSK VarilRix vaccines) and therefore potentially contribute to vaccine attenuation.

To investigate this, we performed comparative analyses of vaccine batches produced by GSK (VarilRix) and Merck (VariVax and ZostaVax), as well as the Biken working seed stock (itself derived from the vOka/Biken seed virus). Our findings include the identification of 137 core SNPs that are shared by all vaccine batches and the Biken working seed stock (which includes 40/42 of the previously reported variant sites). We also demonstrate that while the heterogeneity of vaccine populations differs markedly between GSK and Merck preparations, variation between batches of vaccine from the same manufacturer is minimal. Finally, we examine whether differences in the vaccine allele frequencies at shared variant sites could potentially result in differences in vaccine efficacy.

## MATERIALS AND METHODS

### Vaccine collection.

Three batches of the VariVax live attenuated VZV vaccine preparation were collected in 2008 (VVAG [USA]), 2010 (VV10 [United Kingdom]), and 2012 (VV12 [United Kingdom]). One batch of ZostaVax was obtained in 2015 (ZV15 [United Kingdom]). Two batches of GSK were collected in 2008 (VL08 [United Kingdom]) and 2013 (VL13 [United Kingdom]). One batch of the Oka/Biken working seed stock was kindly provided by Y. Gomi in 2014. In all cases, batch and lot numbers were used to verify all vaccines as independent preparations (Table 1).

**TABLE 1:** Overview of vaccine batches used in this study

Sample ID	Vaccine preparation	Yr of manufacture	Batch no.	Source country
VV10	VariVax	2010	NL13110	United Kingdom
VVAG	VariVax	2009	1526X	USA
VV12	VariVax	2012	G007544	United Kingdom
ZV15	ZostaVax	2015	L029213	United Kingdom
VL08	VarilRix	2008	A70CA882A/AD06A508A	United Kingdom
VL13	VarilRix	2013	Unavailable	United Kingdom
Biken	Oka/Biken working seed	2008^*a*^	M6002	Japan

a Used for vaccine manufacturing from 2011 to 2015.

### Library construction, targeted enrichment, and sequencing.

Total DNA was extracted from each sample using the QiaAMP DNA minikit (Qiagen) according to the manufacturer's instructions, and DNA was quantified using a Qubit HS DNA assay (Invitrogen). Sequencing libraries were constructed using 200 ng of input DNA in accordance with the standard SureSelect XT v1.5 (Agilent) protocol, and enrichment for VZV sequences was performed as described previously (23). Samples were sequenced across several Illumina MiSeq runs according to availability.

### Genome assembly and variant calling.

Adaptor sequences and low quality bases were trimmed from read data using Trim Galore (http://www.bioinformatics.babraham.ac.uk/projects/trim_galore/). Data sets were aligned against VZV strain Dumas (NC_001348) using BWA (24). The resulting alignments were processed using Picard (http://broadinstitute.github.io/picard) and the GATK Toolkit (25) to remove duplicate reads and perform local realignment. SAMTools (26) was subsequently used to generate pileup files for each sample. A consensus sequence for each data set was called using a custom PERL module. Variant profiling for each data set was performed using VarScan v2.2.11 (27) with the following parameters: a basecall quality of ≥20, a read depth of ≥50, and independent reads supporting minor allele of ≥2 per strand. In addition, variant calls showing a directional strand bias of ≥0.85 were excluded from further analyses. Consensus sequences were generated for each rash sample, but iterative repeat regions (OriS, R1, R2, R3, R4, and R5) and the terminal repeat region were trimmed before further analysis.

### Accession number(s).

All sequencing data are available from the European Nucleotide Archive under study accession number PRJEB14639.

## RESULTS

### Comparative sequencing of Merck, GSK, and Biken vaccines reveals a core set of 137 SNPs.

**FIG 2:**
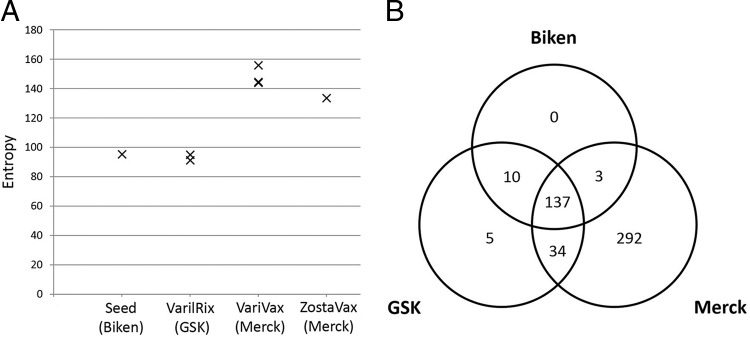
(A) vOka population heterogeneity is highest in Merck preparations (VariVax and ZostaVax). Entropy was used as the measure of the population diversity within a sample, with greater entropy indicating greater diversity. (B) A total of 137 core SNPs were identified in all vaccine batches, one or more of which are assumed to contribute to attenuation. SNPs that are not shared between all batches (or are unique to a manufacturer) are presumed to have been gained or lost during production of the individual manufacturers master and/or working seed stocks. Note that the greater heterogeneity observed in Merck vaccine batches (see panel A) is derived from the additional 292 biallelic sites present only in VariVax and ZostaVax batches.

**FIG 3:**
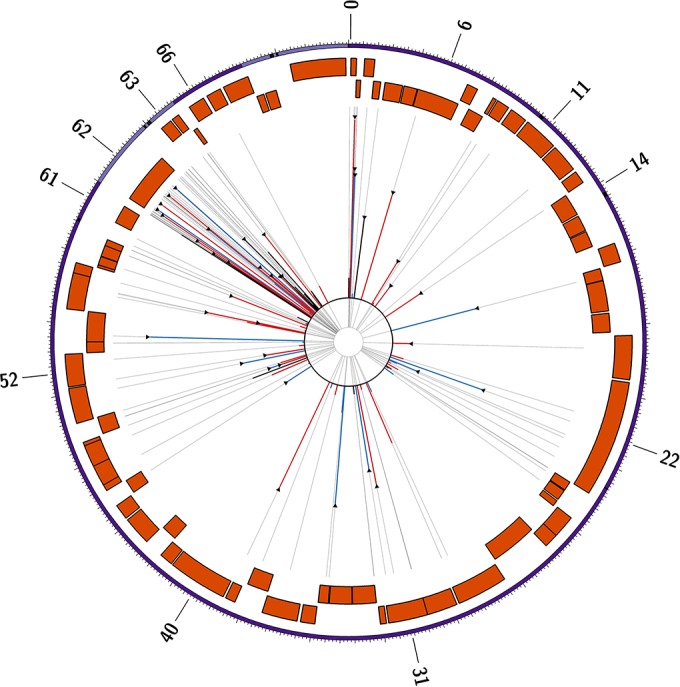
Circos plot showing the location of the core 137 variant sites common to all vOka-derived preparations. Vaccine allele frequencies are shown by the length of the red (nonsynonymous), blue (synonymous), and black (noncoding) lines, all overlaid on a backbone (gray) indicating the exact position of the SNP. Triangular headers indicate sites previously identified by Gomi and coworkers (17). The outer band shown the canonical genome structure (UL-IRL-US-TRL) with the IRL and TRL shown in lilac and the R1, R2, R3, R4, R5, and OriS repeat regions shown in black. Forward and reverse coding ORFs are shown as orange blocks. Relevant ORF numbers are indicated around the outside of the plot.

Deep sequencing of seven batches of vaccine was performed using sequence libraries enriched for VZV DNA (22, 23). Full details of the vaccine batches sequenced are shown in Table 1. After quality control of the sequence data, paired-end reads were aligned to the VZV reference strain Dumas (NC_001348 [28]) and subjected to deduplication and local realignment, after which intrasample population profiles were produced. Using entropy as a measure of population diversity, we observed an increased heterogeneity in Merck vaccine batches when contrasted against GSK vaccine batches and the Oka/Biken working seed, both of which are comparably diverse (Fig. 2A). We subsequently compiled a list of 481 variant sites (SNPs at which two alleles are present) that are detected in at least some genomes obtained from each of the vaccine batches“ (see Table S1 in the supplemental material) and revealed a conserved core of 137 SNPs that are present in all vaccine batches and the Oka/Biken working seed (Fig. 2B). No unique variable sites were identified in the Oka/Biken working seed, whereas just five unique variable sites were identified in the GSK vaccine batches. In contrast, 292 unique variable sites were identified in the Merck vaccine batches, which presumably reflects the differences in the vaccine production process. Of the 137 core sites (Fig. 3; see also Table S2 in the supplemental material), 57 encoded nonsynonymous changes and 27 encoded synonymous changes, while 53 encoded mutations in noncoding regions. A total of 20.54% of the core sites were located within ORF62, while no other open reading frame (ORF) contained more than 4.38% (median, 0.73%) of the core sites.

At only 32/137 sites was the vaccine allele present at >50% frequency in one or more preparations (14 nonsynonymous, 11 synonymous, and 7 noncoding mutations). This increases to 52/137 sites at >25% and 77/137 sites at >10%. Of note, at only a single site (106262, R958G in ORF62) was the vaccine allele fixed at 100% (i.e., there is no evidence of any haplotypes in any vaccine mixture carrying a different allele). In addition, the vaccine allele was present at >98% at three additional sites, two of which encoded synonymous mutations (105705, A1143; 108111, P341), with one nonsynonymous SNP (107252, S628G), all in ORF 62. A total of 40/42 of the original SNP differences reported between the Oka/Biken vaccine (lot V-76) and pOka (9) were accounted for within the set of core sites. Those absent comprised two sites encoding synonymous mutations at 84091 and 106710. The former is absent in all Merck batches sequenced to date but present in Biken and GSK batches, while the latter is absent in all batches, suggesting that this was a polymorphism specific to the Oka/Biken vaccine preparation used in that study.

### Minimal batch-to-batch variation exists in GSK and Merck vaccine batches.

**FIG 4:**
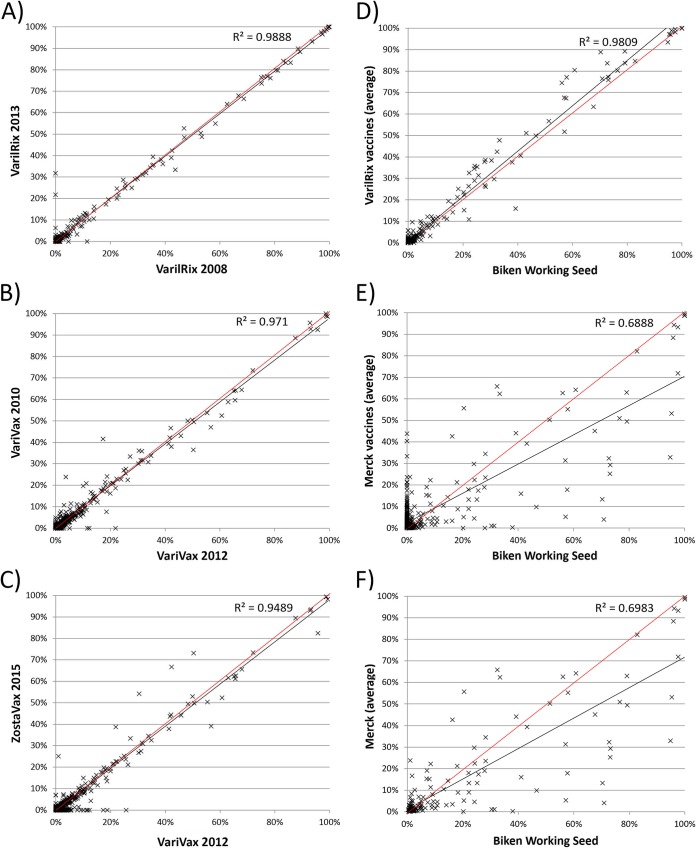
(A to F) Comparison of vOka vaccine allele frequencies between two batches of VarilRix (2008 and 2013, 238 SNPs) (A), between two batches of VariVax (2008 and 2012, 466 SNPs) (B), between one batch of VariVax (2012) and one batch of ZostaVax (2015) (466 SNPs) (C), between GSK VarilRix (average) and Biken working seed (188 SNPs) (D), between Merck VariVax/ZostaVax (average) and Biken working seed (475 SNPs) (E), and between Merck VariVax/ZostaVax (average) and Biken working seed (F) at the core 137 sites only. Best-fit trendlines are shown in black and are compared to the “(0, 1)” line, shown in red. The axes indicate the percent frequency of the vOka allele in that preparation.

Batch-to-batch variation between two VarilRix batches (2008 and 2013) and two VariVax batches (2008 and 2012) is minimal, as demonstrated by the *R*^2^ correlation values (0.988 and 0.971, respectively) (Fig. 4A and B). Similarly, a high *R*^2^ correlation score (0.945) was observed between the 2012 batch of VariVax and the 2015 batch of ZostaVax (Fig. 4C). While up to 26 variant sites were unique to any given batch of vaccine, none of these exceeded 32% frequency (median, 1.58%).

### Merck vaccine batches are distinct from GSK vaccines and the Biken working seed vaccine.

**FIG 5:**
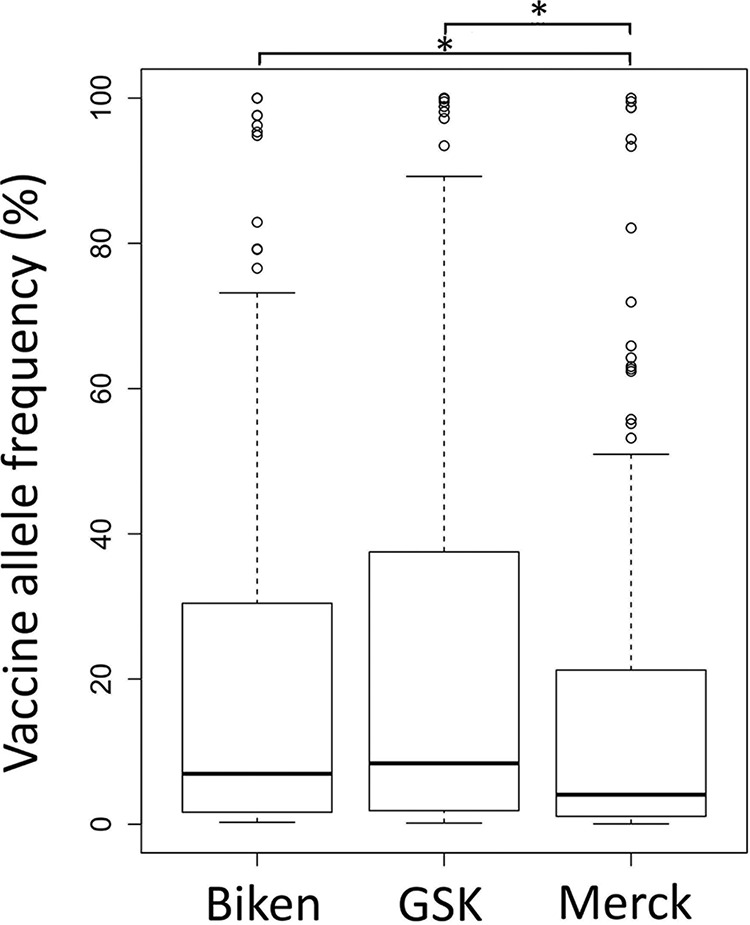
Box-and-whisker plots showing differences in vaccine allele frequency distributions between the Biken working seed and the averages of GSK vaccines and Merck vaccines. The box represents data lying between the lower and upper quartiles (25% to 75%), while the whiskers indicate the nominal range of the data (inferred from the upper and lower quartiles). The median vaccine allele frequency is indicated by the solid bar. Values lying beyond the nominal range of the data are shown as open circles. Significant differences (Mann-Whitney U test, *P* < 0.05) are indicated by an asterisk (*).

A high correlation (*R*^2^ = 0.981) in vaccine allele frequencies was also evident when we compared vaccines produced by GSK and the Biken working stock (Fig. 4D). By comparison, the correlation between Merck vaccines and the Biken working stock (and by extension, the correlation between Merck and GSK vaccines) was markedly lower, whether considering all sites (*R*^2^ = 0.689, Fig. 4E) or just the core sites (*R*^2^ = 0.697, Fig. 4F). Focusing purely on the core variant sites (Fig. 5), the mean vaccine allele frequencies were significantly lower in Merck vaccines than in GSK vaccines (*P* = 0.02, Mann-Whitney U test) and the Biken working seed (*P* = 0.04).

## DISCUSSION

Commercial VZV Oka vaccines, aimed at preventing chickenpox and shingles, are available from three manufacturers (GSK, Biken, and Merck). All three preparations are derived from the live attenuated vOka seed stock originally produced in 1972. The first comparative sequencing study comparing the wild-type virus (pOka) and the attenuated virus strain (Biken vOka) revealed 42 SNP differences (9) but was hampered by the use of older and less sensitive sequencing technologies. By combining Illumina sequencing with SureSelect enrichment, we have confirmed the presence of 40 of these SNPs and identified a further 97 SNPs to obtain a core set of 137 SNPs that are common to all vaccine preparations and therefore likely to have been present in the original attenuated virus stock. For most of these, the vaccine allele frequency is less than 20% However, six sites (560, *130R, ORF0; 105544, V1197A; 105705, A1143; 106262, R958G; 107252, S628G; and 108111, P341 [all ORF62]) are consistently fixed (106262) or near fixation (>90%) in all three preparations, three of which (560, 106262, and 107252) have been shown to be important for attenuation, although heterogeneity (3% wild-type allele frequency) at position 106262 in one batch of VariVax vaccine has been reported using a TA cloning methodology (19). Another 22 are present at frequencies of >20% in all three. Two of these sites, both located in intergenic regions (positions 105010 and 110379), have not previously been reported. Four of the core sites (560, *130R, ORF0; 105356, I1260V; 106262, R958G; and 107252, S628G [all ORF62]) are shared with VZV strain Ellen (see Table S2 in the supplemental material), which has been shown to be attenuated for growth in SCID-hu mice skin explants (11), although, it is not known whether the Ellen phenotype is clinically attenuated. One site, position 560 (*130R), is also shared with the Korean SudaVax preparation, which is derived from a Korean wild-type strain using the same methodology as that used for the original vOka (29). However, no other vaccine-associated mutations have been described for SudaVax, and there are no data confirming that this vaccine is attenuated either *in vitro* or clinically.

Vaccine allele frequencies at the core sites differ between vaccine preparations from different manufacturers, with vaccine alleles being present at a higher frequency for 67.8% of the core sites in the GSK and Biken vaccines versus the Merck vaccine. This finding supports previous data showing higher vaccine allele frequencies for 15 loci in the GSK vaccine compared to the Merck vaccine (20) and probably reflects differences in manufacturing process. For instance, the GSK vaccine underwent a series of terminal dilutions during production of the Oka/GSK seed stock (Fig. 1), resulting in a bottleneck that removed many low-frequency vaccine alleles from the population.

However, more than 82% of the 292 variants specific to Merck vaccines were present in two or more batches. This, together with the finding that at least 158 of the 292 variant alleles have previously been observed in vaccine rash cases after inoculation with VariVax vaccines, suggests that most of these mutations were acquired during the production of the working seed lot that currently forms the basis of all batch production and not during vaccine batch production itself (22). Thus, batch-to-batch variation is low for both Merck and GSK vaccine preparations (Fig. 4A and B), reflecting the success of the tight controls on working practices for live vaccine production. Importantly, most of the mutations seen in vaccine preparations from all three manufacturers are preexisting, and new mutations appear to arise rarely and remain at low frequency. Moreover, the conservation in variant populations extends between vaccine preparations (Fig. 4C) from Merck used for vaccinating against chickenpox (VariVax) and those used to prevent herpes zoster (ZostaVax), despite a 14-fold difference in PFU between the preparations.

None of the three preparations have been directly compared for vaccine efficacy or adverse events. However, higher seroconversion rates have been reported for the Merck (VariVax) vaccine compared to the GSK (VarilRix) vaccine (30, 31). Similarly, a comparative study of vaccine effectiveness following an outbreak at a nursery reported a higher risk of breakthrough disease in individuals who had received the GSK vaccine compared to those who had received the Merck vaccine (32). Thus, the higher core vaccine allele frequencies in the GSK vaccine may have resulted in slight reduction of immune responses and protection. This suggests that loci other than the six sites that are fixed or near fixation in both preparations may contribute to vaccine attenuation. However, although there are considerable data on these available for the Merck preparation, data for adverse events following the GSK and Biken preparations are limited or absent, and therefore no conclusions can be drawn.

In summary, we have produced a definitive catalogue of the mutations present in vOka vaccine preparations from three manufacturers. Although differences between the vaccines do exist, there appear to be a substantial number of vaccine sites that are shared, including one at which the vaccine allele is fixed and five at which the vaccine allele is near fixation (>90% frequency) in all three. We propose that these are critically important for attenuation. However, limited data from clinical and serological studies provide strong support for the notion that other sites in the genome are also likely to contribute (11). Despite differences between vaccine preparations, we confirm that there is little batch-to-batch variation for two of the vaccine preparations tested, suggesting that new mutations arising during manufacture of the vaccine batches being few in number and at low frequency are unlikely to impact vaccine safety and efficacy.

## Supplementary Material

Supplemental material
