# Supplementary material for: Deep Sequencing of Distinct Preparations of the Live Attenuated Varicella-Zoster Virus Vaccine Reveals a Conserved Core of Attenuating Single-Nucleotide Polymorphisms
Source: J Virol. 2016 Sep 12;90(19):8698–704. doi: 10.1128/JVI.00998-16 (PMC5021409; doi:10.1128/JVI.00998-16)
Supplement: Supplemental material [file supp_90_19_8698__index.html]

Deep Sequencing of Distinct Preparations of the Live Attenuated Varicella-Zoster Virus Vaccine Reveals a Conserved Core of Attenuating Single-Nucleotide Polymorphisms — Supplemental material 

# Deep Sequencing of Distinct Preparations of the Live Attenuated Varicella-Zoster Virus Vaccine Reveals a Conserved Core of Attenuating Single-Nucleotide Polymorphisms

## Supplemental material

- Supplemental file 1 -

  Table S1 (vOka (vaccine) allele frequencies across all SNPs.)

  Table S2 (vOka (vaccine) allele frequencies across the core 137 SNPs.)

  XLSX, 67K
